# Optimized FDG-PET/MRI protocol reveals metabolic predictors of long-term survival in pancreatic cancer patients

**DOI:** 10.3389/fonc.2024.1448444

**Published:** 2024-11-27

**Authors:** Freimut D. Juengling, Ines Valenta-Schindler, Alin Chirindel

**Affiliations:** ^1^ Department of Oncology, Cross Cancer Institute, University of Alberta, Edmonton, AB, Canada; ^2^ Medical Faculty, University Bern, Bern, Switzerland; ^3^ Department of Nuclear Medicine, University Washington at St. Louis, St. Louis, MO, United States; ^4^ Department of Nuclear Medicine, University Hospital Basel, Basel, Switzerland

**Keywords:** pancreatic neoplasms, positron emission tomography computed tomography, magnetic resonance imaging, fluorodeoxyglucose F18, prognosis, survival analysis, tumor biomarkers, PET/MRI

## Abstract

**Purpose:**

To optimize and assess an abbreviated dual time-point 18-Fluor-Deoxyglucose (FDG)-Positron Emission Tomography (PET)/Magnetic Resonance Imaging (MRI) protocol for predicting patient outcomes in pancreatic cancer.

**Methods:**

70 patients (47 pancreatic cancer, 23 chronic pancreatitis) underwent hybrid PET/MRI with dual time-point PET/CT at 60 and 84 minutes post-injection. Metabolic indices (MI) were calculated from Standardized Uptake Value (SUV) changes (SUVmin, SUVmean and SUVmax). Multivariate analysis was performed on PET, MRI, laboratory, and histologic data. Top predictors were used for survival analysis.

**Results:**

MI SUVmax, thresholded at 11%, was the best outcome predictor, distinguishing high-risk (2year (2y)-Overall Survival (OAS) 32%, 5y-OAS 14%, 10y-OAS 8%) and low-risk groups (2y-OAS 76%, 5y-OAS 32%, 10y-OAS 23%). Tumor size, CBD obstruction, and infiltrative disease had lower predictive value.

**Conclusions:**

Metabolic indices from abbreviated dual time-point FDG-PET/MRI can differentiate pancreatic malignancy from pancreatitis and predict outcomes, outperforming other indices. This protocol offers a valuable diagnostic tool for characterizing pancreatic lesions and predicting outcomes based on imaging criteria.

## Introduction

Despite increased research efforts and promising therapy trials over the past decade, the prognosis for pancreatic cancer has not significantly improved compared to other gastrointestinal malignancies. Additionally, the incidence of pancreatic cancer has markedly risen over the last two decades. In 1990, pancreatic cancer was responsible for 200,000 deaths worldwide and was the 11th most frequent cancer in women and the 12th in men (1). By 2016, the global death toll had doubled to 405,000 (2). In 2022, pancreatic cancer was estimated to be the second most frequent gastrointestinal cancer in the United States (U.S)., surpassed only by colonic cancer, and accounting for 8% of total cancer deaths, ranking as the fourth leading cause of cancer-related mortality in both men and women (3).

Unfortunately, the symptoms of pancreatic malignancies are nonspecific and often appear late in the disease progression. Consequently, up to 80% of patients are diagnosed at stage II-III, by which time the cancer has either spread or progressed locally to a degree that makes surgical intervention unfeasible. In such cases, treatment options are limited and primarily palliative, typically involving aggressive chemotherapy regimens, sometimes in combination with radiation therapy. These treatments generally result in survival gains of approximately one year, as indicated by retrospective studies (4, 5). Even for stage I disease cases that are suitable for surgical intervention, the overall five-year survival rate remains between 12% and 15% (6–8). The primary curative treatment for stage I pancreatic cancer is surgical resection, which has evolved from traditional open surgery to minimally invasive techniques. These minimally invasive approaches have the potential to reduce postoperative recovery time and associated complications. However, both open and minimally invasive or robot-assisted pancreatic surgeries carry significant risks of severe complications (9–11). Therefore, a risk-adjusted treatment strategy should be employed whenever feasible, incorporating a comprehensive preoperative evaluation with Computed Tomography (CT) or MRI scans to guide treatment planning.

### Preoperative diagnostic challenges and role of imaging

Ideally, preoperative planning mandates confirming a histologic diagnosis through biopsy. However, technical hurdles impeding biopsy procedures and the presence of heterogeneous lesions, which can lead to sampling errors, may result in scenarios where the definitive diagnosis is only established during surgery. Any advancements in imaging techniques capable of predicting the final diagnosis will ultimately aid in tailoring the surgical approach, potentially reducing postoperative complications and morbidity (12).

Nevertheless, differentiating between benign and malignant pancreatic lesions solely through imaging modalities remains a diagnostic challenge. Despite employing sophisticated clinical imaging protocols such as multi-phase contrast-enhanced CT, multi-parametric MRI, endoscopic ultrasound, PET/CT, and the more recent PET/MRI, these imaging procedures consistently demonstrate low specificity in distinguishing pancreatic malignancies from acute or chronic inflammatory pancreatic conditions (13–17). Clinical research protocols incorporating kinetic metabolic analyses from dynamic PET studies, established over a decade ago, have demonstrated superior performance in distinguishing between malignant and inflammatory pancreatic lesions (13, 15, 18–20) and predicting outcomes when compared to traditional PET, CT, and even multiparametric MRI (21, 22). Despite these advantages, the dynamic PET imaging protocols utilized in these studies have not been widely adopted in clinical practice. This is primarily due to the need for mathematical kinetic modeling and continuous image acquisition of a restricted field of view, in addition to whole-body imaging. This resulted in imaging protocols extending up to 120 minutes, thereby occupying scanner time that could otherwise accommodate six to ten consecutive patients. The recent introduction of total-body scanners with large axial fields of views and significantly enhanced scanner sensitivity characteristics, when combined with MR imaging, holds the potential to substantially improve pancreatic tissue characterization through the use of kinetic descriptors (23). However, this advanced and costly technology is still in its early stages of introduction and remains limited in availability.

The objective of this study was to assess an abbreviated and simplified, semiquantitative hybrid imaging protocol for prediction the outcomes of pancreatic lesions utilizing standard clinical scanner technology within a busy, clinical setting.

## Materials and methods

### Patients

Patient consent and Institutional Review Board (IRB) requirements according to good clinical practice (GCP) and the Declaration of Helsinki of 2000 were met prior to any patient study. Over a span of 9 years, a total of 70 consecutive patients (40 male, 30 female) undergoing diagnostic evaluation for localized pancreatic lesions were prospectively enrolled in the study and subsequently followed for a minimum of 10 years. Exclusion criteria encompassed laboratory results indicative of acute pancreatitis, a history of prior malignancy, previous surgical intervention, as well as prior chemotherapy or radiotherapy treatments. Median age at the time of diagnostic procedure was 71.2 years (range 42-82 years). The medical and demographic records of patients encompassed age, gender, serum Carcinogenic Antigen 19-9 (CA-19-9) and bilirubin levels, lesion size and location, histological diagnosis, presence or absence of jaundice, TNM stage for malignant cases, including tumor grading based on World Health Organization (WHO) criteria for digestive system tumors classification [cf. reference (15)], along with treatment information.

### PET/MR imaging

Every patient underwent hybrid PET/MR imaging, which involved whole-body PET/CT using a dedicated clinical scanner (Siemens Biograph 40 HIREZ TRUE-D, Siemens Healthineers, Erlangen, Germany) with a non contrast-enhanced low-dose CT scan (90mAs, 110kV, CAREDOSE). This was followed by a contrast-enhanced diagnostic CT scan if a previous contrast-enhanced CT was not available, and then an abdominal MR. All patients fasted for a minimum of 6 hours before the PET scan and underwent blood glucose testing right before the injection of 5 Megabequerel/Kilogram (MBq/Kg) FDG. Patients with established non-insulin-dependent diabetes were instructed to take their oral diabetes medication at least one hour before the examination. In cases where blood glucose levels at the scheduled PET appointment exceeded 10.1 mmol/l, the examination was either postponed or blood glucose was normalized by administering a suitable dose of fast-acting human insulin intravenously. In these cases, the injection of FDG was halted until subsequent blood glucose testing indicated the beginning of a rise in glucose levels following the initial decrease. The PET protocol included a whole body scan 60 minutes post injection (4 minutes emission time per bed, 4-5 beds per examination), immediately followed by a single bed acquisition of the abdomen (4 minutes emission time). Within one week of PET/CT imaging, a whole-body MRI was conducted, followed by a dedicated contrast-enhanced MRI of the abdomen. (Siemens 1.5T Verio, Siemens Healthineers, Erlangen, Germany). Each patient underwent either fine needle biopsy, exploratory surgery, or definitive resection, resulting in cytological or histological diagnoses. PET/CT and MRI scans were analyzed using a dedicated oncology workstation (Siemens Multimodality Workplace) with manufacturer-provided proprietary software (TRUE-D, Siemens Healthineers, Erlangen, Germany). The evaluation process involved the following steps: inter- and intramodality co-registration of whole-body PET/CT and MRI scans, contrast-enhanced CT scans, second time-point PET/CT scans, and post-contrast abdominal MRI scans; fusion of the co-registered images; and regional analysis of pancreatic lesion characteristics. To conduct regional analysis, areas of contrast enhancement or solid appearance in lesions (or solid-appearing areas in the absence of contrast enhancement) were delineated as irregular three-dimensional (3-D) regions-of-interest (ROIs) on the patient’s MRI by three experienced investigators (FDJ, IV, and AC). These delineated regions were then transferred onto the two co-registered PET/CT datasets to align suspected tumorous regions with their corresponding regional metabolic activity. For regional analysis, contrast enhancing or (in case of missing contrast enhancement) solid appearing lesion areas were independently delineated by three experienced investigators (FDJ, IV and AC) as irregular 3-D ROIs on the patient’s MRI and copied onto the two co-registered PET/CT data sets to match suspected tumorous areas and their respective regional metabolism. For each region of interest and time-point SUV were quantified as the maximum SUV (SUVmax), mean SUV (SUVmean), and minimum SUV (SUVmin) to account for potential metabolic heterogeneity within the tumor. Metabolic indices (MI) were derived by calculating the percent change in SUV (SUV%) using the formula: 100*((SUV at Timepoint 2 / SUV at Timepoint 1) − 1)%. Inter-observer variations in the SUV measurements were also assessed. To mitigate potential observer-induced bias, the mean values of the three investigators’ respective SUV measures were utilized for further analysis.

### Statistical analysis

As the primary endpoint, overall survival (OAS) was defined as the duration in months from the date of diagnosis to the date of death from any cause. Multivariate analysis was conducted to identify independent predictors of survival. For SUV measures proving to be a predictor of survival, hazard ratios and p-values were calculated for incremental values by performing a Cox proportional hazards regression analysis, and the significance and effect size of each threshold was compared using the Akaike Information Criterion (AIC) to compare models. Receiver Operating Characteristics (ROC) analyses were then performed to determine the accuracy of cutoff/grouping values for variables predictive of OAS, followed by univariate analysis of OAS for the best-performing cutoff values. Additionally, Chi-square and Fisher exact tests were used to compare frequencies between groups. Survival time was estimated using the Kaplan-Meier method, and differences in survival between groups were compared using a log-rank test. Multivariate Cox’s proportional hazards regression was applied to assess whether metabolic indices provided additional predictive information on survival, with a p-value <0.05 indicating statistical significance. A heatmap indicating hazard ratios and p-values for the 20 top performing variables was calculated. Statistical analysis was performed using MEDCALC software version 14.8.1 (www.medcalc.org).

### Patient enrollment and diagnosis

Seventy consecutive patients underwent hybrid early dual time-point FDG-PET/MRI. Pancreatic adenocarcinoma was confirmed in 47 patients, while 23 were diagnosed with chronic pancreatitis. Among those with adenocarcinoma, 35 (74%) had surgical resections, and 12 (26%) received biopsies only due to advanced disease identified by PET/MRI.

### Follow-up and survival analysis

At analysis time, 41 patients had died, with a median follow-up of 157.5 months (range 140-204 months), a mean OAS of 40.1 months and a median OAS of 26.5 months (range: 3.4-185 months). Patient demographics and clinical characteristics are summarized in **Table 1**.

**Table 1:** Demographical and baseline characteristics: patients with diagnosis of pancreatic cancer.

	Number of patients (n=47)	%
Age, years, median (range)	69.00 (46-88)	
Sex		
Male	20	42.5
Female	27	57.5
Tumor size [cm], median (range)	3.0 (0.5-9.0)	
Histologic differentiation		
Well (G1)	7	14.9
Moderate (G2)	23	48.9
Poor (G3)	17	36.2
T classification		
T1-2	10	21.2
T3	23	49.0
T4	14	29.8
N classification		
N0	22	46.8
N1	25	53.2
TNM stage		
Ia/b	6	12.8
IIa	5	10.6
IIb	16	34.0
III	10	21.2
IV	10	21.2
**CA19-9 level [U/mL], median (range)**	73 (0.0-71854.0)	
≤200 (U/mL)	29	62
>200 (U/mL)	18	38
**SUVmax, Median (range)**	3.55 (1.81-12.10)	
MI SUVmax, median (range)	13.7 (-23.6-49.3)	
MI SUVmean, median (range)	12.9 (-18.1-41.0)	
MI SUVmin, median (range)	15.6 (-17.8-82.2)	
CHD obstruction		
Yes	22	46.8
No	25	53.2
Location		
Pancreatic head	36	76.5
Pancreatic body	9	19.1
Pancreatic tail	2	4.3
Jaundice		
Yes	21	44.6
No	26	55.3
Resection margin		
Negative	35	74.5
Positive	12	25.5
Adjuvant treatment		
Surgery	11	23.4
Surgery + Chemotherapy	6	12.8
Surgery + Chemoradiotherapy	16	34.0
Surgery + Radiotherapy	2	4.3
Chemotherapy	4	8.5
Chemoradiotherapy	8	17.0

### Results

**Figure 1:**
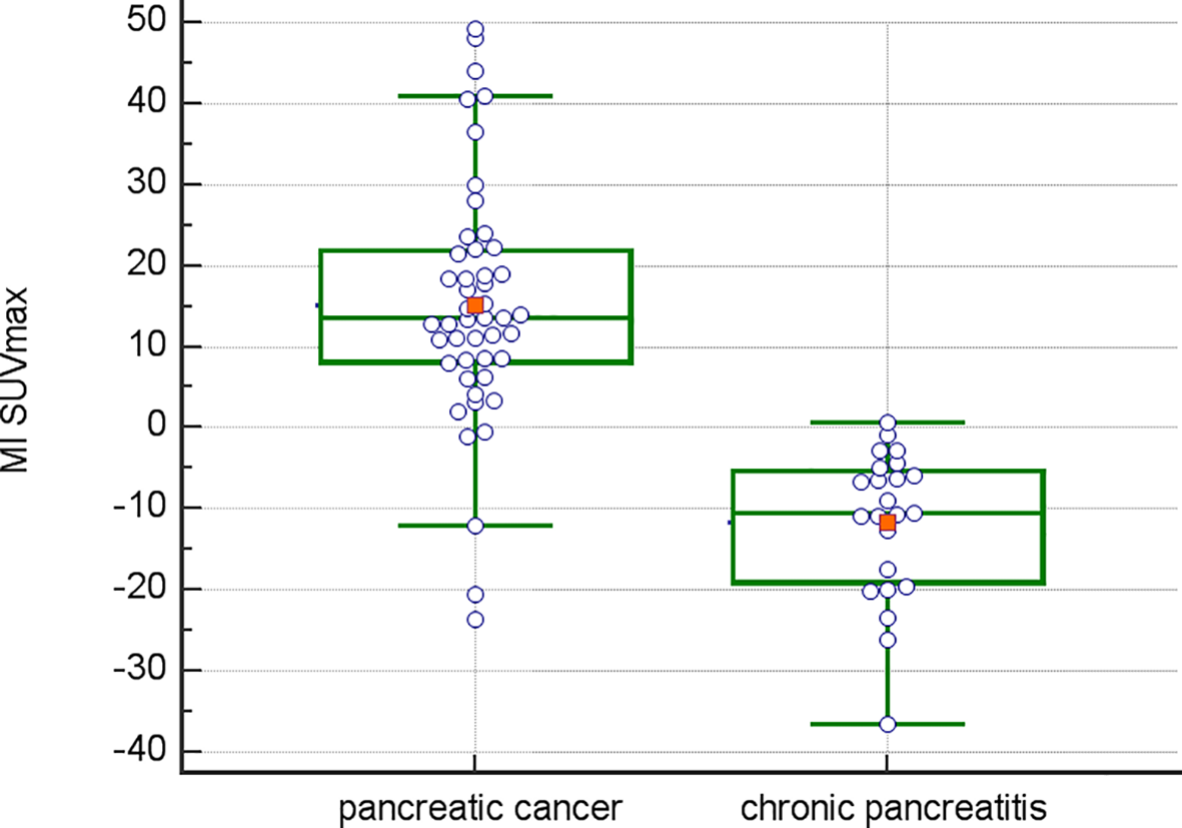
Data comparison graph for MI SUVmax in pancreatic cancer vs. pancreatitis.

**Figure 2:**
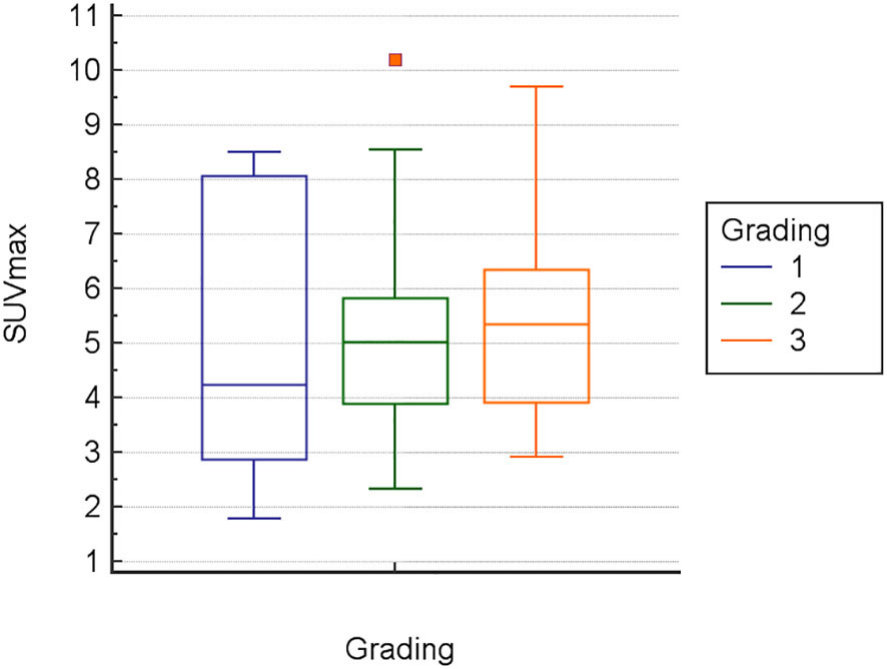
Data comparison graph for SUVmax vs. tumor grade in pancreatic cancer.

Inter-observer variance for SUV measurements was minimal: 2.2% for SUVmin, 2.8% for SUVavg, and 2.1% for SUVmax. The mean Metabolic Index for SUVmax (MI SUVmax) was significantly different between pancreatic cancer (14.7) and chronic pancreatitis (-11.6), *p*<0.0001 (**Figure 1**). Demographical and baseline characteristics for patients with diagnosis of chronic pancreatitis are given in **Table 2**. SUVmax did not differentiate between tumor grades 1-3 (**Figure 2**).

**Table 2:** Demographical and baseline characteristics: patients with diagnosis of chronic pancreatitis.

	Number of patients (n=23)	%
**Age** [years], median (range)	63.8 (43-86)	
Sex		
Male	14	60.9
Female	9	39.1
**Lesion size [cm], median, (range)**	3.0 (0.8-7.3)	
**SUVmax, median, (range)**	2.49 (0.8-7.3)	
MI SUVmax, median, (range)	-10.4 (-36.6-2.7)	
MI SUVmean, median, (range)	-9.6 (-30.4-4.69)	
MI SUVmin, median, (range)	-11.7 (-40-3.75)	
Location		
Pancreatic head	36	76.5
Pancreatic body	9	19.1
Pancreatic tail	2	4.3
Jaundice		
Yes	21	44.6
No	26	55.3

### Multivariate analysis

A multivariate proportional-hazards Cox regression model included clinical and imaging parameters such as SUVmax, metabolic tumor volume, age, TNM classification, and CA-19-9 levels. Key predictors of poor outcomes were M-stage, tumor location, CA-19-9 levels, and MI SUVmax. The optimal MI SUVmax cutoff (>11) was determined for incremental value increases w-using the Akaike Information Criterion and confirmed by ROC curve analysis.

### Hazard ratios and P-values

**Figure 3:**
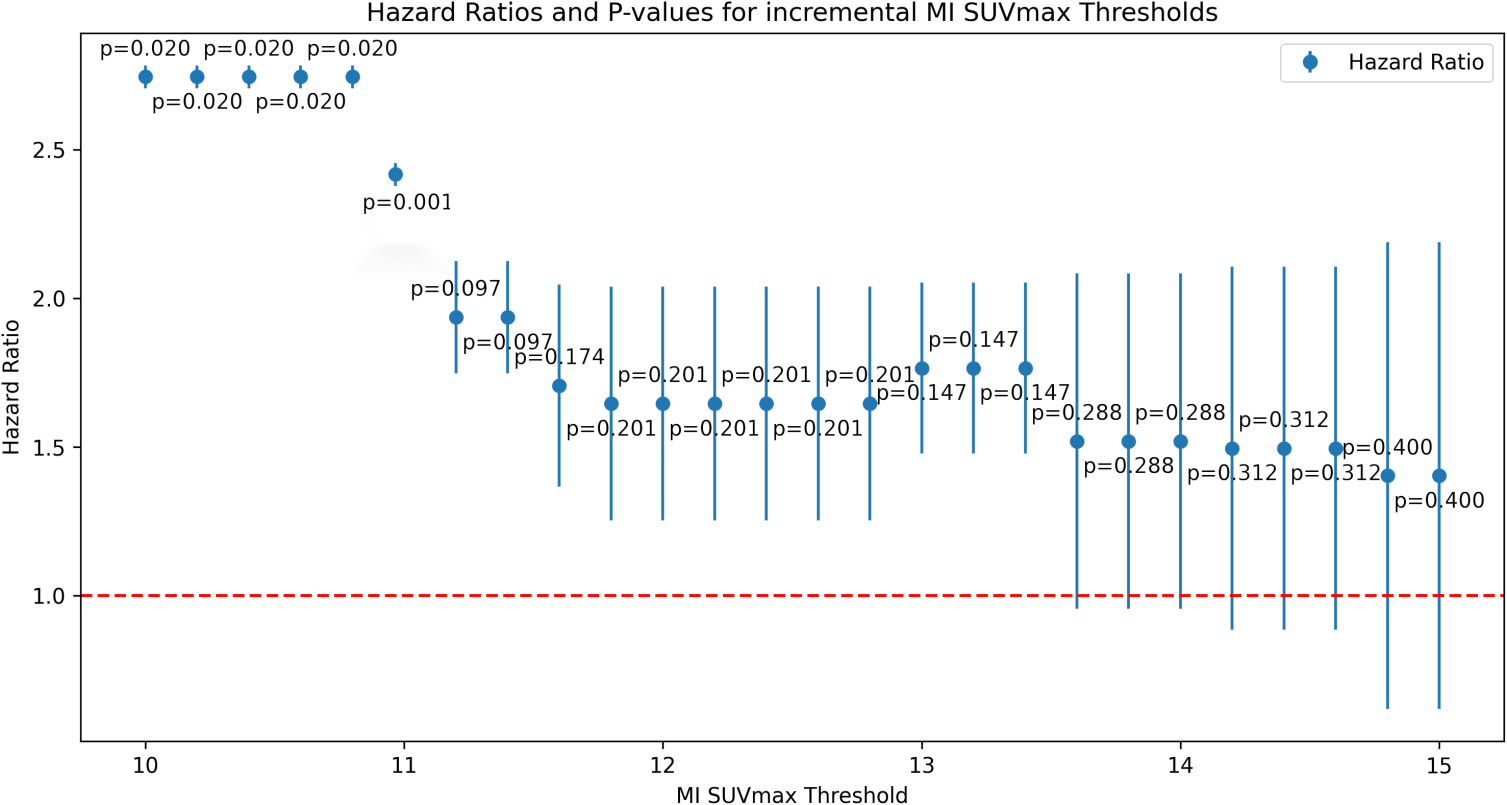
Hazard ratios and p-values for incremental MI SUVmax thresholds.

**Figure 3** displays hazard ratios and p-values for incremental MI SUVmax values. A heatmap of the top variables is presented in **Table 3**.

**Table 3:** Heatmap of hazard ratios and p-values (sorted by hazard ratios).

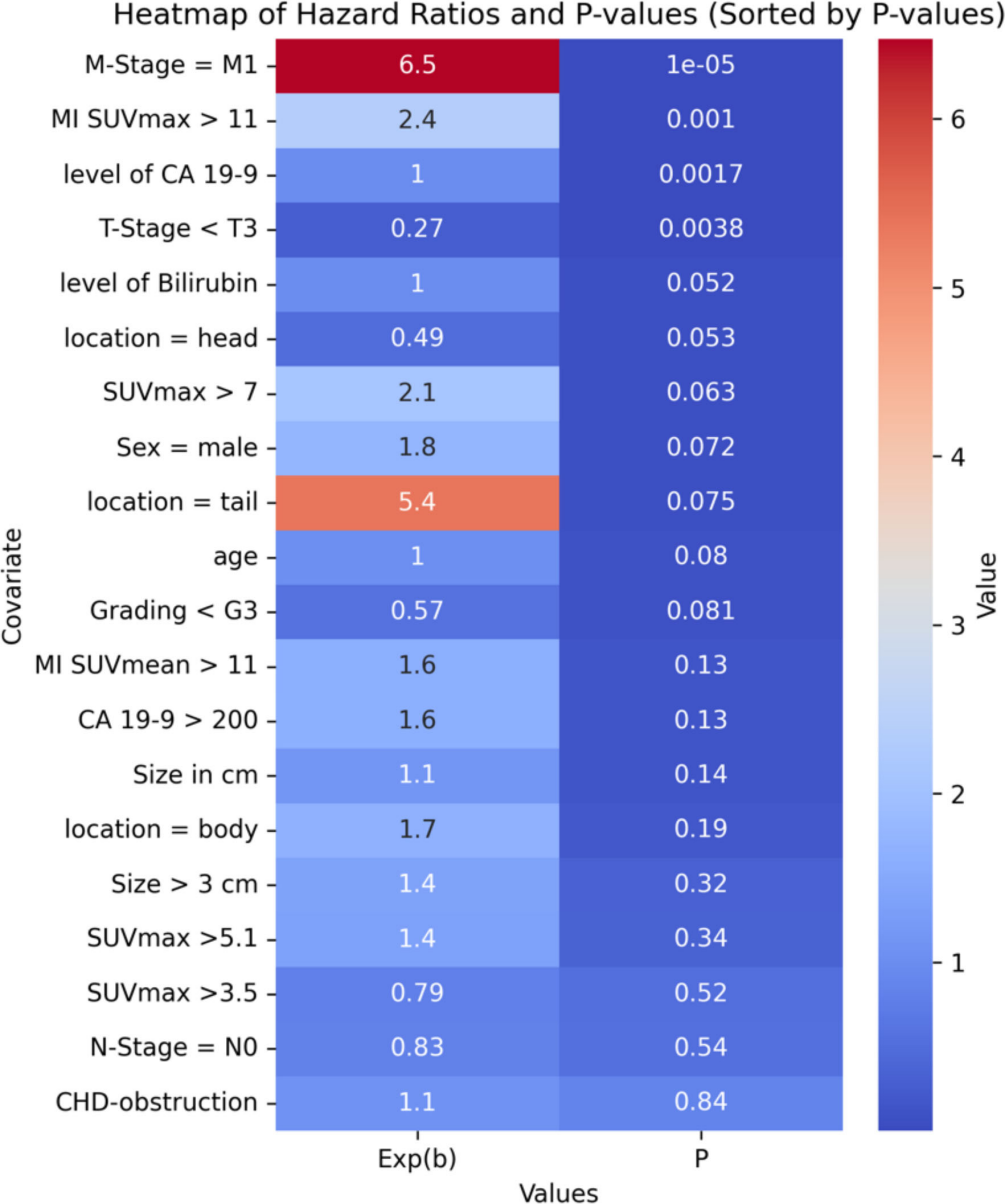

### Group comparisons

Patients were divided based on MI SUVmax >11. No significant differences in baseline characteristics were found between groups.

### Univariate analysis

Univariate analysis showed that T-stage >T2M0, tumors in the pancreatic body or tail, and MI SUVmax >11 were associated with shorter overall survival (OAS). Other factors like sex, age, tumor grade, CA19-9 levels >200 U/mL, and CHD obstruction were not significantly associated with OAS (**Table 4**).

**Table 4:** Univariate analysis of overall survival (OS).

Variables		Number (total=47)	Mean OS (months)	Median OS (months)	95% CI	*p value*
**Age**	<60	4	73.3	8.3	4-8	*0.229*
	≥60	43	41.6	21.4	17-38	
**Sex**	Male	20	29.9	16.6	9-27	*0.068*
	Female	27	47.3	31.0	19-46	
**Tumor size**	<3	23	50.1	26.7	30-73	*0.310*
	≥3	24	38.3	20.0	14-27	
**T classification**	T1-2	10	79.2	67.6	32-71	0.002*
	T3-4	37	29.7	19.1	15-26	
**N classification**	N0	22	41.6	21.4	19-34	*0.540*
	N1	25	40.1	21.0	10-32	
**M stage**	M0	37	56.6	27.0	20-43	<0,0001*
	M1	10	11.7	9.4	8-16	
**Location**	Pancreatic head	36	55.0	26.2	17-34	0.019*
	Pancreatic body	9	23.0	20.2	16-27	
	Pancreatic tail	2	9.6	9.4	9-10	
**Grading**	Well/Moderate (G1+G2)	29	52.6	27.0	21-43	*0.076*
	Poor (G3)	18	30.5	13.8	9-20	
**CA19-9**	≤200	30	56.5	27.0	19-32	*0.126*
	>200	17	28.8	17.1	8-34	
**SUVmax**	≤3.5	10	63.6	31.5	26-43	*0.172*
	>3.5	37	36.8	20.0	16-31	
**MI SUVmax**	<11	16	66.6	42.7	27-67	0.001*
	≥11	31	33.0	19.1	15-22	
**CHD obstruction**	no	25	43.3	22.3	16-28	*0.842*
	yes	22	47.8	20.2	14-34	

Statistically significant findings are marked with *.

### Prognostic factors

Cox regression identified M-Stage >1 and MI SUVmax >11 as independent prognostic factors (p<0.001 and p=0.001, respectively). A MI SUVmax ≤11 predicted better survival rates at 2 years (76%), 5 years (32%), and 10 years (23%), compared to a MI SUVmax >11.

### Survival analysis

**Figure 4:**
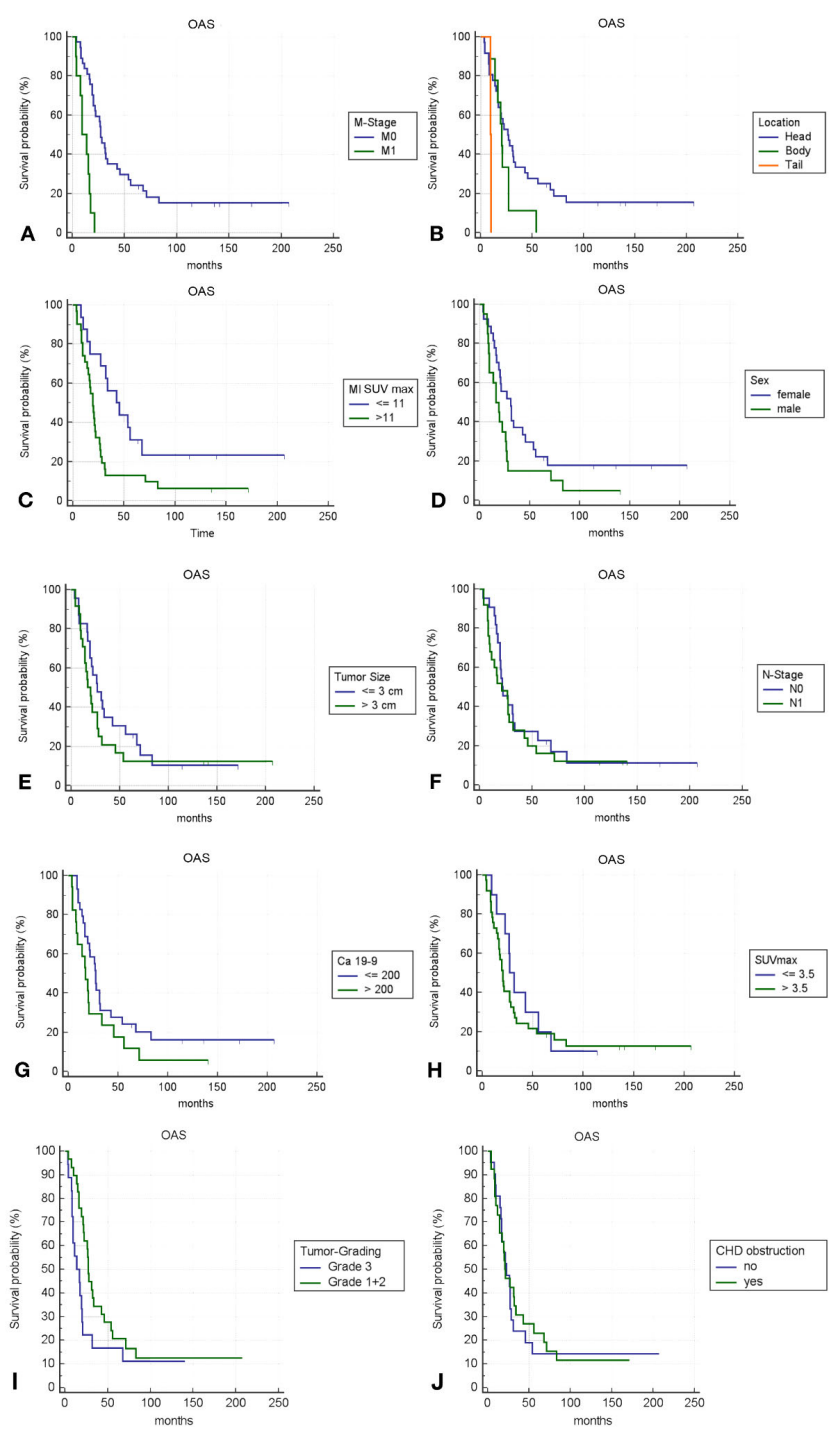
**(A–J)** Kaplan-Meier survival curves for different variables; p-value <0.05 is only fulfilled for **(A–C)**.

Kaplan-Meier survival analyses were conducted to assess the prognostic significance of various previously identified variables (**Figure 4**). Among these, only M-Stage, anatomical tumor location, and Metabolic Index (MI) SUVmax demonstrated statistically significant differences in survival between groups using the log-rank test. MI SUVmax, in particular, was a strong predictor, distinguishing between a high-risk group with an overall survival (OS) rate of 32% at 2 years, 14% at 5 years, and 8% at 10 years, and a low-risk group with an OS of 76% at 2 years, 32% at 5 years, and 23% at 10 years.

### ROC analysis

**Figure 5:**
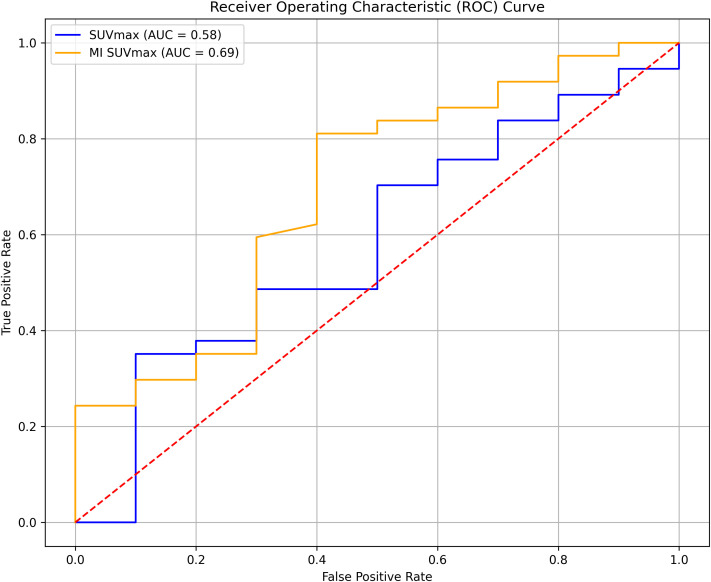
Receiver Operating Characteristic (ROC) curve for SUVmax and MI SUVmax.

The ROC analysis showed that MI SUVmax had better predictive capability for survival outcomes than SUVmax, with AUC values of 0.69 and 0.58, respectively (**Figure 5**). Although neither parameter demonstrated exceptional predictive power, MI SUVmax proved to be the more effective predictor in this analysis.

## Discussion

The present study aimed to evaluate semiquantitative dual time-point hybrid FDG-PET/MR measurements as indicators of potential malignancy in pancreatic lesions and predictors of outcomes in patients with pancreatic carcinoma. While it is well established that elevated glucose consumption plays a crucial role in cancer progression, and FDG can serve as an indicator of glycolysis in malignant tissue, the visual intensity of FDG uptake does not always accurately reflect the *in-vivo* metabolic activity of the cells generating the FDG PET signal. This discrepancy arises because the locally measured radioactivity over time represents a composite signal that integrates the activities from phosphorylated intratumoral, non-phosphorylated intravascular, and interstitial radiotracer sources (24).

Time course analysis in quantitative FDG-PET investigations of pancreatic lesions, utilizing dynamic image acquisition over at least 90 minutes, has demonstrated the ability to differentiate between malignant and inflammatory diseases, as well as normal pancreatic metabolism (13, 15, 18–20). However, to our knowledge, no systematic data have been available to date to determine its prognostic value in predicting overall survival.

In an effort to establish an optimal, streamlined protocol for clinical imaging, the time-activity curves from the original dynamic data in (18) were meticulously re-analyzed. The goal was to identify the minimal time lag between two single measurements of tumor activity that could effectively differentiate between benign and malignant diseases while simplifying the data acquisition process. Based on the slopes of the time-activity curves at different time points, an initial time point of 64 minutes post-injection (corresponding to when the pancreas would typically be imaged during a standard whole-body PET scan) and a second time point at 84 minutes post-injection were identified. These two time points were selected because they were expected to have large enough differences in the slopes of the time-activity curves to potentially differentiate between benign and malignant pancreatic diseases. To test this hypothesis and determine the prognostic value of dual time-point hybrid PET/MR in pancreatic cancer, this histologically controlled study was initiated to investigate abbreviated time-point imaging in a large, prospective cohort of patients with pancreatic lesions.

Corroborating data from a prior report (25), metabolic indices differed significantly between pancreatic malignant and inflammatory disease (**Figure 1**). The hypothesis that an increase in regional metabolism over a short 20-minute interval (increase in metabolic index) could differentiate between malignant and inflammatory diseases, was confirmed in all patients, with a positive predictive value (PPV) of 100%, negative predictive value (NPV) of 47%, accuracy of 95%, sensitivity of 63%, and specificity of 100%. In contrast, single time-point imaging at the initial time point had a PPV of 81%, NPV of 55%, accuracy of 73%, sensitivity of 19.6%, and specificity of 95% when a cutoff of 7 was chosen for SUVmax, as suggested as best predictive parameter by ROC analysis of thresholds. Interestingly, there were 3 cases of adenocarcinoma with non-mucinous pathology, where the MI was negative. Histopathological findings in these patients revealed predominantly inflammatory disease with smaller areas of malignancy within. Conversely, in five malignant lesions that initially showed no visually increased FDG uptake above background at the initial time point (the typical time point for routine clinical imaging), a subsequent increase in SUVmax of more than 11% (MI >11) was observed at the second time point. The MI in the segmented tumor volume significantly exceeded the MI in the surrounding normal pancreatic tissue (data not shown).

It is well known that the plateau of intracellular FDG metabolic trapping, due to FDG not being a substrate for glucose metabolism downstream of hexokinase, is typically reached approximately 90 minutes post-injection (26–28). Additionally, it has been shown that imaging later than 90 minutes post-injection may further improve tumor-to-background contrast and identify more lesions (29). However, for the sake of simplicity and patient workflow, clinical practice generally adopts a post-injection uptake period of only 60 minutes prior to imaging, as a compromise between the total duration of the procedure and diagnostic efficacy. The potential loss of sensitivity and efficacy due to this less-than-optimal clinical practice can be partially compensated by incorporating regionally defined second time-point measures at time points closer to the plateau phase of intracellular FDG trapping.

Another notable finding was, that in 11 cases comprising small lesions and lesions with complex or partially cystic anatomy, regional metabolic changes were only accurately determined when ROIs were specifically delineated based on lesion contours on hybrid PET/MRI. The traditional method of defining spherical ROIs centered on visually perceived hypermetabolic lesions seen on PET/CT images alone, which included the entire lesion and surrounding tissue, failed to show significantly increased MIs in these cases. In complex and very small lesions, this may be attributed to increased bias due to local metabolic inhomogeneity, with partial volume effects masking regional metabolic changes and initial peak values not necessarily located in the tissue of interest (e.g., in adjacent vascular structures). This may also have contributed to the finding, that SUVmax, in our cohort, did not differentiate between tumor grade 1-3 (**Figure 2**).

Regional analysis based on lesion delineation on hybrid PET/MRI proved to be both feasible and reproducible in a clinical setting, resulting in excellent inter-observer variance of less than 3%.

In the multivariate Cox regression model, MI SUVmax >11 was a key predictor of poor outcomes, with a hazard ratio indicating a substantial increase in risk. The statistical significance of this threshold was confirmed by ROC curve analyses, underscoring its robustness as a prognostic tool.

Compared to other potential thresholds, MI SUVmax >11 demonstrated superior predictive accuracy for overall survival. While previously referenced thresholds for SUVmax, such as 3.5 and 5.1 (30, 31) did not show significant prognostic value in our patient cohort, the MI SUVmax threshold at 11 consistently differentiated between outcomes effectively (compare **Table 3**).

In our patient cohort, the previously cited thresholds for SUVmax of 3.5 and 5.1 did not demonstrate significant prognostic value, as reported in earlier studies (30, 31), compare **Table 3**.

In our analysis, M-stage was confirmed as well-known predictor of poor outcomes, with a hazard ratio of 6.4730. However, the interpretation of this finding may be limited due to the relatively small number of patients presenting with metastases at diagnosis. Additionally, tumors located in the pancreatic tail were associated with a worse prognosis, although this association did not reach statistical significance.

Clinically, the identification of MI SUVmax >11 as an optimal cutoff may provide valuable guidance for treatment planning and risk stratification. Patients exceeding this threshold are associated with significantly shorter overall survival, highlighting the need for more aggressive management strategies.

The use of hybrid PET/MRI in this study provided several key advantages over traditional PET/CT for accurately assessing regional metabolic changes in pancreatic lesions and predicting prognosis:

### Improved delineation of complex and small lesions:

In 11 cases with small or partially cystic lesions, metabolic changes could only be accurately determined when regions of interest (ROIs) were specifically delineated based on the lesion contours seen on the MRI component of the hybrid PET/MRI. The superior soft tissue contrast of MRI allowed for more precise ROI placement compared to the traditional method of drawing spherical ROIs on the non-contrast low-dose CT images typically acquired in PET/CT alone. This is especially important for complex lesions where metabolic inhomogeneity and partial volume effects can mask regional changes if the ROI includes surrounding normal tissue, which would specifically affect SUVmean values.

### Exclusion of non-tumorous hypermetabolic foci:

Another advantage of MRI is the ability to better characterize incidental hypermetabolic foci in the vicinity of the pancreas as non-tumorous based on their MRI appearance. With PET/CT alone, such foci could potentially be mistaken for further tumor extension and be included wrongly into the tumor ROI. The improved soft-tissue contrast of MRI helps avoid this pitfall.

### Detection of subtle tumor infiltration:

MRI is also superior for detecting tumor infiltration into surrounding tissues compared to CT. This again allows for more accurate tumors delineation.

In summary, although MI SUVmax was identified as the best prognostic predictor, the incorporation of MRI in this study played a vital role in accurately quantifying this metric in complex cases. The superior soft tissue contrast of MRI enabled precise tumor delineation, exclusion of confounding non-tumorous hypermetabolic foci, as well as exclusion of diluting the metabolic index through partial volume effects, which is particularly important when considering the potential advantages of using SUVmean values instead of SUVmax. SUVmax, being a single-pixel value, is more susceptible to noise and may not accurately represent the overall tumor metabolism, especially in heterogeneous lesions. In contrast, SUVmean, which averages the metabolic activity over the entire tumor volume, may provide a more robust assessment of tumor metabolism. However, accurate calculation of SUVmean heavily relies on precise tumor segmentation, which is greatly facilitated by the superior soft tissue contrast of MRI. By improving the interpretation of metabolic information from PET, the hybrid PET/MRI approach laid the foundation for exploring the potential benefits of using SUVmean as an alternative or complementary prognostic marker to SUVmax.

It can be expected that the clinical use of integrated PET/MRI scanners will further enhance regional analysis by minimizing partial volume effects caused by potential motion-related subtle misregistration between the two exams and possible changes due to disease progression or declining inflammatory changes within the inter-modality time interval.

### Optimized protocol

The additional time and effort required for dual time-point imaging and analysis using the presented abbreviated protocol are minimal and can be easily integrated into the existing clinical workflows of many oncologic imaging centers. In a systematic workflow, reporting already includes co-registration and matching of all existing imaging modalities for image reading, and reporting of SUV is a recommended part of the regular report in oncologic PET imaging (32). Therefore, the additional time needed for an extra SUV measurement is negligible. Consequently, for patients with suspicious pancreatic lesions, the proposed abbreviated dual time-point hybrid FDG-PET/CT/MRI protocol offers an excellent diagnostic option. It aids in characterizing pancreatic lesions, predicting individual patient outcomes, and can be seamlessly incorporated into or adapted to existing local clinical imaging protocols using standard imaging equipment.

With the increasing availability of integrated PET/MRI scanners, processing time can be further minimized, and issues related to image misregistration and inter-modality abdominal motion can likely be overcome, potentially enhancing the ability to accurately define prognostic criteria in small and heterogeneous pancreatic lesions. Additionally, new total-body scanner technology, with its dramatically increased signal-to-noise ratio in dynamic data, may contribute to even better diagnostic performance.

### Conclusion

In conclusion, the utilization of hybrid PET/MRI for analyzing regional metabolic changes over time has proven to be a valuable tool in enhancing the diagnostic evaluation of pancreatic lesions by providing additional predictors of patient outcomes and reliably differentiating between chronic pancreatitis and pancreatic carcinoma. A key finding of this analysis is the identification of the MI SUVmax threshold at 11 as a reliable and clinically relevant imaging marker for predicting survival in pancreatic cancer patients. This imaging marker effectively distinguishes between low-risk and high-risk patient groups, offering crucial prognostic information. The low-risk group demonstrates a 2-year overall survival (OAS) of 76%, a 5-year OAS of 32%, and a 10-year OAS of 23%, while the high-risk group shows a 2-year OAS of 32%, a 5-year OAS of 14%, and a 10-year OAS of 8%.

The integration of this marker into clinical practice has the potential to significantly enhance decision-making processes and ultimately improve patient outcomes, which can be obtained through the implementation of the proposed abbreviated protocol of hybrid early dual time-point PET/MRI, offering an efficient and effective approach to pancreatic cancer assessment and prognosis.

### Strengths and limitations

Strength of this study is the prospective design of systematic data acquisition and analysis including a follow-up time interval sufficient to define 10-year OAS. A further strength of the concept presented is that it is directly transferrable to the workflow of currently available, integrated PET/MRI systems (proposed workflow given in Appendix I). Limiting is the relatively small number of total patients, not allowing for meaningful subgrouping of histopathologically differing cases (e.g. mucinous vs. non-mucinous vs. mixed appearance, Intraductal Pancreatic Mucinous Neoplasia (IPMN), where bias to small size effects in histopathologic heterogeneous disease can not fully be accounted for.

## Data Availability

The raw data supporting the conclusions of this article will be made available by the authors, without undue reservation.
